# Long Term Molecular Epidemiology of Methicillin-Susceptible *Staphylococcus aureus* Bacteremia Isolates in Sweden

**DOI:** 10.1371/journal.pone.0114276

**Published:** 2014-12-05

**Authors:** Gunlög Rasmussen, Stefan Monecke, Ole Brus, Ralf Ehricht, Bo Söderquist

**Affiliations:** 1 Department of Infectious Diseases, Örebro University Hospital, Örebro, Sweden; 2 School of Health and Medical Sciences, Örebro University, Örebro, Sweden; 3 Alere Technologies GmbH, Jena, Germany; 4 Institute for Medical Microbiology and Hygiene, TU Dresden, Dresden, Germany; 5 Clinical Epidemiology and Biostatistics, Örebro University Hospital, Örebro, Sweden; 6 Department of Laboratory Medicine, Clinical Microbiology, Örebro University Hospital, Örebro, Sweden; 7 Faculty of Medicine and Health, Örebro University, Örebro, Sweden; University of Edinburgh, United Kingdom

## Abstract

*Staphylococcus aureus* is one of the major pathogens that causes bacteremia; therefore, it is important to understand the long-term molecular epidemiology of *S. aureus* bacteremia infections. In particular, little is known about the population structure of methicillin-sensitive *S. aureus* (MSSA) compared to that of methicillin-resistant *S. aureus*. We investigated potential changes in the MSSA molecular epidemiology in Örebro County, Sweden, from 1980 through 2010. 400 MSSA bacteremia isolates, the first 100 isolated each decade from 1980 through 2010, were retrospectively identified and analyzed regarding assignment to clonal complexes (CCs), presence of virulence genes and antibiotic resistant determinants with DNA microarray-based genotyping. 24 different CCs were identified. Most isolates (80%) belonged to 6 predominant lineages. Of those, the number of isolates assigned to CC5 and CC15 increased, and those assigned to CC8, CC25, and CC30 decreased. The most prevalent clone, CC45, did not show a significant change in prevalence during the study period. A change in prevalence was observed for some of the virulence genes, mainly attributed with their association to certain CCs. With the exception of the common *blaZ* gene (encoding penicillinase), antibiotic resistance genes were only sporadically detected. In conclusion, the MSSA population structure was genetically diverse. We observed decadal changes in assignments to five predominant clones, and corresponding changes in the prevalence of some virulence genes linked to CC affiliation. In light of the restrictive antibiotics prescriptions and extensive infection control procedures in Sweden, antibiotic resistance genes were rarely detected and their prevalence unaffected during the study period.

## Introduction

*Staphylococcus aureus* is an important pathogen and one of the most common causes of bacteremia, with high mortality [1], [2]. *S. aureus* produces several virulence factors, including exotoxins, enzymes such as serine and cysteine proteases, regulating factors, and adhesion proteins, which contribute to its pathogenicity [3], [4].

Some studies have shown an increased incidence of *S. aureus* bacteremia (SAB) [5], [6]. In part, this increase may be explained by increases in general life expectancy, overall morbidity (including immune-compromising conditions), and the use of advanced medical interventions that require invasive devices, like indwelling catheters and prosthetic devices. Furthermore, the extensive misuse of antibiotics worldwide has led to the emergence of resistant pathogens, such as methicillin resistant *S. aureus* (MRSA). This may also contribute to the increased incidence of SAB [7]–[9].

We previously found an association between *S. aureus* invasive disease and bacterial genotypes. Among methicillin-sensitive *S. aureus* (MSSA) strains, we found that certain clonal complexes (CCs); 5, 8, 15 and 25 and specific virulence genes, such as those encoding accessory gene regulator group II (*agr* II), capsule polysaccharide serotype 5 (*cap*5), and some adhesins, were more prevalent in bacteremia isolates than in isolates that colonized the nares [10].

Overall, the molecular epidemiology of MSSA infections is less studied compared to that of MRSA. Although a few previous studies have investigated the molecular epidemiology of MSSA infections over time [11]–[13], few have studied changes in the MSSA population structure over long time periods. The aim of the present study was to investigate potential changes in MSSA molecular epidemiology from 1980 through 2010. With a DNA microarray-based genotyping assay, we analyzed 400 SAB isolates that originated in 1980-81, 1990-91, 2000, and 2010, regarding assignment to CCs, the presence of virulence genes, and the acquisition of antibiotic resistance genes.

## Materials and Methods

### Setting and bacterial isolates

In Örebro County, the estimated population ranged from 270,000-284,000 between 1980 and 2010. The three hospitals in the county were served by one microbiology department. Thus, it was possible to identify all cases of SAB treated at any of the hospitals in the county. According to local routines established from 1980, isolates from all blood cultures that yielded positive results were stored at the Department of Laboratory Medicine, Clinical Microbiology, at Örebro University Hospital.

We retrospectively identified the first 100 consecutive episodes of SAB detected each decade from 1980 to 2010. The corresponding blood culture isolates comprised four comparable study groups. The microbiology database provided information about gender and age at the time of diagnosis, but no other clinical data. Consequently, we could not classify the bacteremia as community-onset, healthcare-associated, or nosocomial. SAB was defined as at least one positive blood culture performed with the Bactec system (Becton Dickinson, USA). No more than one isolate per patient was included.

The *S. aureus* isolates were identified by routine microbiological methods, such as coagulase and DNase tests. Isolates had been stored at −70°C in preservation medium (yeast extract; Difco Laboratories, Sparks, MD, USA; and horse serum added to trypticase soy broth; BBL, Sparks, MD, USA).

### DNA microarray-based genotyping

Genotyping was performed with the Alere StaphyType DNA microarray test (Alere Technologies GmbH, Jena, Germany), which included 333 target sequences that corresponded to approximately 170 distinct genes and their allelic variants. The targets included species markers, capsule types, regulatory loci, genes encoding microbial surface components recognizing adhesive matrix molecules (MSCRAMMs), exotoxins, antibiotic resistance markers, and others. The procedure and the primer and probe sequences have been described previously in detail [14]–[16].

Briefly, *S. aureus* isolates were subcultured on Columbia blood agar overnight at 37°C, and then enzymatically lysed with lysozyme, lysostaphin, and RNAse. Spin columns (Qiagen, Hilden, Germany) were used to prepare RNA-free unfragmented DNA, which served as the template in a multiplex primer elongation. In the next step, amplicons labelled with biotin-16-dUTP were hybridized to the microarray. After conjugation with horseradish-peroxidase-streptavidin, hybridizations were visualized with a precipitating dye. An image of the microarray was acquired with a designated reader (Alere Technologies GmbH, Jena, Germany). Normalized intensities of the spots were calculated based on their average intensities and further analyzed as described previously [15].

Isolates were assigned to CCs or sequence types (STs), defined by multilocus sequence typing (MLST) [17]. Briefly, hybridization profiles were compared automatically to a database of reference strains and isolates previously subjected to MLST [14].

### Statistics

Trends of change per decade in assignment to CCs and prevalence of virulence genes, comparing four equal sized groups of SAB isolates with 100 isolates in each originating from four different decades, were expressed as the incidence rate ratio (IRR), estimated by Poisson regression analysis. The denominator used was the number of isolates per decade and the nominator the number of isolates assigned to a specific clonal complex or carrying a specific virulence gene. The estimated rate indicated the decadal change (i.e., a change of 1.20 indicated a 20% increase over each decade, and a rate of 0.95 indicated a decrease of 5%). P-values<0.05 were considered statistically significant. Statistical analyses were performed with STATA 10.1 software.

## Results

**Figure 1:**
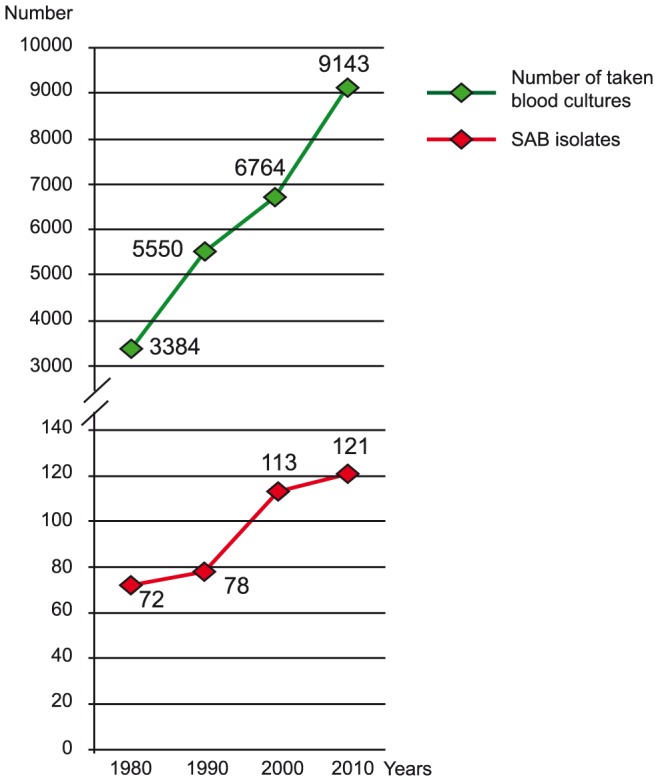
Number of positive blood cultures that yielded *S. aureus* (number of episodes of SAB) in Örebro County, relative to the total blood-culturing rate in concurrent years.

This study included a total of 400 SAB isolates, the first 100 consecutive from each decade (1980, 1990, 2000, and 2010) of the study period. Figure 1 shows the number of positive blood cultures that yielded *S. aureus* (number of episodes of SAB) in Örebro County, relative to the total blood-culturing rate in concurrent years. Since there were less than 100 episodes of SAB during 1980 (n = 72) and 1990 (n = 78), additional isolates from 1981 (n = 28) and 1991 (n = 22) were included to receive equal sized study groups with 100 isolates from each decade.

Characteristics of patients with blood cultures positive for *S. aureus* are shown in Table 1, also subdivided into different age categories for each time period. The number of patients over 75 years increased over time.

**Table 1:** Characteristics of patients with blood cultures positive for *S. aureus*

Characteristic	Total n = 400 (%)	1980-81 n = 100	1990-91 n = 100	2000 n = 100	2010 n = 100
Mean age^1^	62.4	59.7	60.6	64.4	64.6
Age 0–17	30 (8)	6	11	7	6
Age 18–54	76 (19)	25	15	17	19
Age 55–74	133 (34)	37	36	30	30
Age>75	158 (40)	29	38	46	45
Sex (male)	240 (60)	59	57	64	60

1 Age data are missing for 3 patients from 1980-81.

### Antibiotic resistance genes

The *blaZ* gene, encoding penicillinase, was found in 295 isolates (74%). Only 16 *S. aureus* isolates harbored one or more additional antibiotic resistance genes, and the overall prevalence of resistance genes did not increase during the study period (Table 2).

**Table 2:** Presence of antibiotic resistance genes in *S. aureus* isolated from blood cultures, the resistance mechanisms, and the corresponding antibiotics.

				Numbers of isolates with the indicated gene in each time period			
Resistance gene	Protein	Resistance mechanism	Antibiotic	1980-81 n = 100	1990-91 n = 100	2000 n = 100	2010 n = 100
*mecA*	Alternate penicillin-binding protein 2a (PBP2a)	Change in structure of PBP2a	Methicillin	0	0	0	0
*blaZ*	Penicillinase (β-lactamase)	Production of β-lactamase enzymes that hydrolyse the β-lactam ring	Penicillin	73	76	78	67
*vanA*, *vanB*	Ligase enzyme	Change in structure of cell wall peptidoglycan precursors, resulting in altered binding site for vancomycin	Glycopeptides	0	0	0	0
*aacA-aphD*	Acetylation and phosphorylation enzyme	Enzyme that catalyse drug modification	Aminoglycosides	1	0	0	0
*aphA*	Phosphorylation enzyme	Enzyme catalyses drug modification	Aminoglycosides	0	0	0	0
*mupR*	Mupirocin resistance protein	Target modification	Mupirocin	0	0	0	0
*cat*	Chloramphenicol acetyltransferase	Acetylation prevent chloramphenicol from binding to the ribosome	Chloramphenicol	1	1	0	0
*far1*/*fusB*	FusB-proteins associated with fucidic acid resistance	Binds to and modulate the function of the drug target elongation factor G	Fucidic acid	0	0	0	1
*Q6GD50 (fusC)*				0	0	0	3
*tetK*	Tetracycline resistance determinant	Efflux	Glycylcycline	1	2	1	0
*tetM*	Tetracycline resistance determinant	A protein binds to the ribosome, which interferes with binding of tetracycline	Tetracyclines	1	1	0	0
*ermA*	Erythromycin ribosomal methylase	Methylation of 23S rRNA, resulting in target modification on ribosome	MLS-antibiotics^1^	0	2	2	0
*ermB*				0	0	0	0
*ermC*				1	1	1	0

1 Macrolides, Lincosamides, Streptogramins

The *mecA*-gene was not found in any isolate; thus, no isolate was classified as MRSA. Quinolone- and rifampicin resistance were not included in the microarray because these resistance properties are caused in *S. aureus* by mutations in ubiquitous genes.

### Clonal complexes

**Figure 2:**
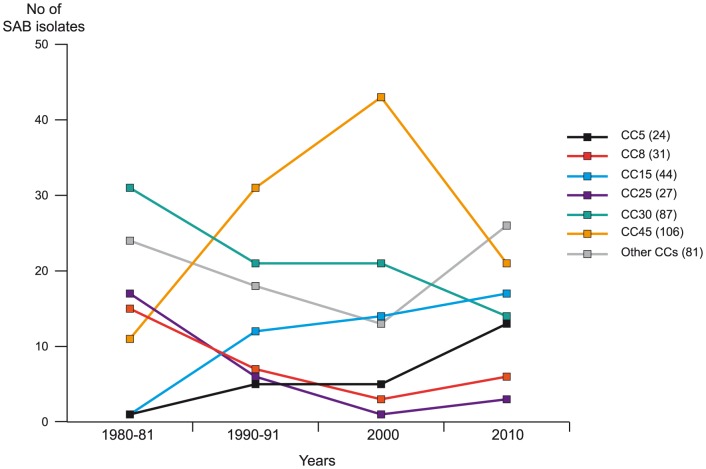
Distribution of SAB isolates assigned to CCs during the study period. Of 6 predominant CCs (CC5, CC8, CC15, CC25, CC30, and CC45), SAB isolates assigned to CC5 and CC15 showed a trend towards an increased prevalence over the study period, and isolates assigned to CC8, CC25, and CC30 declined. The numbers in parentheses indicate the total number of isolates within each CC. CC30 includes CC30 (ST34/42). Other CCs: CC1, CC6, CC7, CC9, CC12, CC20, CC22, CC49, CC50, CC59, CC97, CC101, CC121, CC182, CC188, CC395, CC398, and ST2319.

The distribution of isolates assigned to the different CCs over the study period is shown in Figure 2, given both as the total number within each CCs as well as divided between the four groups of isolates originating from different decades of the study period. The DNA microarray analysis identified a total of 24 CCs. The 6 predominant CCs (CC5, CC8, CC15, CC25, CC30, and CC45) included 319 isolates (80%). CC45 was the most common clone and comprised 106 isolates (27%), followed by CC30, CC15, CC8, CC25 and CC5. The remaining 18 CCs included 1-13 isolates each. Trends of decadal changes in assignment to the major CCs were also calculated using Poisson Regression analysis. The prevalence of CC 45 showed no significant linear change over the study period (IRR = 1.17, p = 0.069). Decadal increases did occur however in the prevalence of CC5 (IRR = 1.96, p = 0.002) and CC15 (IRR = 1.62, p = 0.001), and decreases occurred for CC8 (IRR = 0.66, p = 0.015), CC25 (IRR = 0.44, p<0.001), and CC30 (IRR = 0.79, p = 0.015).

### *Agr* groups

The distribution of *agr* groups in each time period is shown in Table 3. *Agr* group I comprised the largest number of isolates (n = 200; 50%); *agr* group IV alleles were found in only 12 isolates (3%), all assigned to CC50 and CC121. The distributions of *agr* groups I and IV did not change significantly over the study period. Isolates that harbored *agr* group II alleles increased significantly over the years (IRR = 1.36, p = 0.002). Conversely, the prevalence of *agr* group III alleles declined (IRR = 0.81, p = 0.021). One *agr* group might be found in several CCs, but all isolates within a given CC harbored the same *agr* alleles.

**Table 3:** Distribution of *agr* groups (alleles) and presence of virulence genes among SAB isolates from four different time periods.

Virulence gene	Gene product	Numbers of isolates with the indicated gene in each time period				Decadal changes	
*Agr* group		1980-81 n = 100	1990-91 n = 100	2000 n = 100	2010 n = 100	IRR (95% CI)^1^	p-value^2^
*agr* I^3^	Accessory gene regulator I	50	52	52	46	0.98 (0.86–1.19)	0.704
*agr* II^4^	Accessory gene regulator II	11	22	22	33	1.36 (1.12–165)	0.002
*agr* III^5^	Accessory gene regulator III	36	23	22	19	0.81 (0.68–0.97)	0.021
*agr* IV^6^	Accessory gene regulator IV	3	3	4	2	0.94 (0.56–1.55)	0.796
**Exo-polysaccharides**							
*cap*5	Capsular polysaccharide 5	46	24	11	28	0.78 (0.66–0.93)	0.004
*cap*8	Capsular polysaccharide 8	54	76	89	72	1.10 (0.99–1.22)	0.079
**MSCRAMMs**							
*cna*	Collagen binding adhesin	54	62	73	52	1.01 (0.90–1.13)	0.885
*sasG*	*S. aureus* surface protein G	29	36	28	50	1.17 (1.01–1.35)	0.040
**Exotoxins**							
**Leukocidins**							
*lukF, lukS, hlgA*	γ-toxin	100	100	100	100		
*lukF-PV, lukS-PV*	Panton-Valentine leukotoxin	4	1	0	2	0.75 (0.38–1.48)	0.404
*lukD, lukE* 7	Leukocidin D, E component	52	44	34	58	1.02 (0.90–1.16)	0.794
**Haemolysins** 8							
*hla*	α-toxin	80 (8)^14^	99	99 (1)^14^	100	1.04 (0.95–1.14)	0.382
*hld*	δ-toxin	100	100	100	100		
**Exfoliative toxins**							
*etA*	Exfoliative toxin A	5	4	6	2	0.85 (0.55–1.30)	0.449
*etB*	Exfoliative toxin B	0	0	0	1		
*etD*	Exfoliative toxin D	17	6	1	3	0.44 (0.28–0.68)	<0.001
**Enterotoxins**							
*sea*	Staphylococcal enterotoxin A	32	20	13	19	0.80 (0.66–0.97)	0.026
*sea*(N315)^9^	Staphylococcal enterotoxin A, allele from N315	1	7	7	15	1.86 (1.28–2.69)	0.001
*seb*	Staphylococcal enterotoxin B	8 (2)^14^	6 (3)^14^	5 (6)^14^	6 (1)^14^	0.89 (0.63–1.27)	0.530
*sec+sel*	Staphylococcal enterotoxin C+L	10	17	26	17	1.19 (0.96–1.47)	0.110
*sed+sej+ser*	Staphylococcal enterotoxin D+J+R	15	9	4	4	0.60 (0.43–0.85)	0.004
*see*	Staphylococcal enterotoxin E	2	0 (1)^14^	0	0		
*egc-cluster* 10	Staphylococcal enterotoxin G+I+M+N+O+U	71	70	76	57	0.95 (0.85–1.05)	0.331
*seh*	Staphylococcal enterotoxin H	6	2	4	6	1.05 (0.69–1.58)	0.833
*sek+seq*	Staphylococcal enterotoxin K+Q	2	3	1	8	1.63 (0.97–2.73)	0.064
*tst1*	Toxic shock syndrome toxin (TSST)-1	25	18	13	15	0.82 (0.66–1.01)	0.065
**Enzymes**							
*aur*	Aureolysin	100	100	100	100		
*splA* 11 */splB* 12	Serine Protease A, B	52	44	34	58	1.02 (0.90–1.16)	0.079
*splE*	Serine Protease E	78	56	42	54	0.86 (0.77–0.97)	0.011
**Miscellaneous genes**							
*edinB*	Epidermal Cell differentiation inhibitor B	17	6	1	3	0.44 (0.28–0.68)	<0.001
*setC* 13	Staphylococcal exotoxin-like protein	62 (1)^14^	66 (4)^14^	77 (1)^14^	83 (3)^14^	1.11 (1.00–1.23)	0.050

1 Incident rate ratio estimated with Poisson regression analysis.

2 Poisson regression analysis.

3 CC6, CC7, CC8, CC20, CC22, CC25, CC45, CC59, CC97, CC101, CC182, CC188, CC395, CC398, ST2319.

4 CC5, CC9, CC12, CC15, CC49.

5 CC1, CC30, including CC30 (ST34/42).

6 CC50, CC121.

7 Three isolates in CC20 and 6 isolates in CC395 were negative for *lukD*, but positive for *lukE*; results were counted for *lukE*.

8 The haemolysin gene *hlb* was excluded from the analysis on the grounds of poor probe performance, which yielded several ambiguous results.

9 Also known as enterotoxin *sep.*

10 Seven isolates in CC50 showed a partial deletion of the *egc* cluster missing *seg.*

11 Locus tag SACOL1057, GenBank CP000046.1: Position 1063016–1064026.

12 Locus tag SACOL1056, GenBank CP000046.1: Position 1061753–1062934.

13 Locus tag SACOL1970, GenBank CP000046.1: Position 2034319–2035485.

14 Numbers in brackets show the number of ambiguous results, which were not included

Bold values are statistically significant.

### Surface associated capsule polysaccharides

The SAB isolates harbored either capsular polysaccharide (*cap*) genes associated with capsule type 5 (n = 109; 27%) or 8 (291; 73%) (Table 3). Prevalence of *cap* 5 genes showed a declining trend during the study period (IRR = 0.79, p = 0.004). All isolates within a given CC carried the same *cap* genes. Polysaccharide intercellular adhesion (PIA) genes, *icaA*, *icaC*, icaD were harbored by all isolates.

### MSCRAMMs

Of 15 analyzed MSCRAMM genes, only *sasG*, which encodes the *S. aureus* surface protein G (Table 3), showed a change in prevalence over the decades (IRR = 1.17, p = 0.040). The prevalence of this gene mirrors CC affiliations, with predominant clones such as CC5, CC8 and CC15 harboring this gene. The *cna* gene, which encodes collagen binding adhesion, was detected in 241 isolates (60%) (Table 3), and the presence was correlated with the affiliation to certain CCs such as being positive in CC1, CC30 and CC45. Other MSCRAMM genes, including *fnbB*, *sdrD*, and *bbp* were present in 321 (80%), 344 (86%), and 368 (92%) isolates respectively; *ebh* was found in 390 (98%) isolates, which represented all except the isolates in CC22 (n = 8) and 2 of the isolates in CC30. The genes *map*, *fib*, and *fnbA* were also harbored by almost all isolates (98–99%). The remaining MSCRAMM genes, *clfA*, *clfB*, *ebps*, *eno*, *sdrC*, and *vwb* were found in all isolates.

### Exotoxins

The presence of genes encoding leukocidins, haemolysins, exfoliative toxins, enterotoxins, enzymes, and other miscellaneous genes are shown in Table 3. Panton-Valentine Leukocidin genes (*lukF-PV, lukS-PV*) were found in only 7 isolates, which originated in different decades. Although the genes that encoded exfoliative toxins (*etA*, *etB*, *etD*) were also rare, a declining trend could be observed for *etD*-positive isolates (IRR = 0.44; p<0.001), which all belong to CC25. The same trend was shown for the *edinB* gene, which encodes the epidermal cell differentiating inhibitor, since this gene was found together with the *etD* gene. Indeed, both genes are known to be co-localized on one transposon (see for instance GenBank AB057421.1). *EdinA* was absent and *edinC* was found in only one isolate.

Of the enterotoxin genes, the *egc*-cluster was most frequently detected; found in 274 (69%) isolates. The prevalence of the *sea* gene showed a decline (IRR = 0.80, p = 0.026). Of *sea*-positive isolates, a majority were assigned to CC1, CC8 and CC30. Moreover the *sed*/*sej*/*ser* genes showed a declining trend (IRR = 0.60, p = 0.004), while the prevalence of *sea* (N315) increased (IRR = 1.86, p = 0.001), with positive isolates mainly belonging to CC5 and CC12. The *tst* gene was found in 71 (18%) isolates, mainly among isolates from CC30 and CC395.

The serine protease gene, *splE* showed a modest decline over the years (IRR = 0.86, p = 0.011). Other protease genes (*splA, splB*) came together with the leucocidin genes (*lukD, E*) since they are located on the same pathogenicity island. Their prevalence did not differ significantly during the study period, and among predominant clones, these genes were mainly harbored by isolates assigned to CC5, CC8, CC15, and CC25. The *setC* (*selX*, locus and gene position in Table 3) was found in 288 (74%) isolates, and its prevalence increased slightly with time (IRR = 1.11, p = 0.050).

## Discussion

The SAB isolates included in this study (all MSSA) showed great genetic heterogeneity. Some clones were predominant (CC5, 8, 15, 25, 30, and 45) and present throughout the study period. The greater genetic diversity among MSSA isolates in relation to MRSA, has been shown before [13], [18]–[20]. The dominant CCs found in the present study were consistent with the MLST database and previous published works from the US and Europe [21]–[24]. Our current findings that CC45 included the largest number of isolates, followed by CC30, confirmed our recently published data from the same geographical setting [10]. In this study [10], isolates assigned to CC45 were distributed among nasal carriage and invasive isolates in about the same proportion, while CC30 seemed to be associated with nasal carriage. On the contrary, CC5, CC8, CC15, and CC25 dominated among invasive isolates, which could indicate a more invasive potential of these clonal lineages with ability to cause bacteremia without initial colonization.

Although the main aim of the present study was to analyze the MSSA population structure over time. We found decadal changes in the clonal structures; the prevalence of CC5 and CC15 increased, and the prevalence of CC8, CC25, and CC30 declined. Previous studies investigating the MSSA molecular epidemiology over time are few. By characterizing MSSA isolates in Portugal over a 19-year period, Taveres et al found one of the predominant clones to be present during the whole study period, other clones were found intermittently, and a few seemed to be related with MRSA epidemic clones [12]. Other studies have mainly been limited to a shorter time-period, in which they were not able to find any temporal changes in clonality [11], [13].

In Sweden, as in other parts of northern Europe, *S. aureus* isolates rarely display resistance to antimicrobial agents, which is supported by data in our study. This might imply that the isolates have been exposed less extensively to antibiotics compared to the exposure in many other parts of the world.

Apart from presence of the *blaZ* gene, which did not became more common over time, few isolates harbored additional antibiotic resistance genes. None of the isolates harbored the *mec*A gene. Although an increase in the number of MRSA cases has been reported in Sweden [25], the spread of infections due to MRSA seems to be under control, and few cases of MRSA bacteremia occur. Overall, the prevalence of MRSA is low in Sweden with approximately 2,500 new cases annually (according to statistics from the Public Health Agency of Sweden). The MRSA found in our low endemic area are heterogeneous and diverse displaying numerous genetic backgrounds. The most common CCs are 80, 8, 5, and 1. About 50% of the MRSA isolates are domestic and 65% are community-acquired. However, the known geographic background of some of the CCs found suggest a multiple and random importation of MRSA from epidemic regions into Sweden. Compared to countries with higher prevalence of MRSA, where MRSA epidemic clones seem to affect the MSSA molecular epidemiology [12], [26], this does not seem to be the situation in Sweden.

Sweden has a history of restrictive antibiotic use, both for humans and livestock; moreover, rather extensive infection control procedures are practiced in clinical medicine. Consequently, the isoxazolyl penicillins have remained the first choice of treatment for *S. aureus* infections, including bacteremia. Even, in selective cases with SAB without confirmed beta lactamase, benzyl penicillin could be considered.

The aminoglycoside resistance genes *aadA-aphD* and *aadD* were found in one single isolate only, so that this observation does not preclude the use of aminoglycosides as an additive treatment in case of a septic patient. Sporadic isolates harbored the *erm* genes, which encode resistance to macrolides, lincosamides and streptogramins, and some harbored tetracycline resistance genes (*tetM* and *tetK*). Even at higher frequencies, the tetracycline resistance genes remain a limited clinical problem. First, tetracycline is not the drug-of choice for treating *S. aureus* infections. Second, isolates that harbor these genes remain sensitive to tigecycline [27].

Over the study period, the number of isolates assigned to *agr* group II tended to increase in parallel with a decrease in the number of isolates assigned to *agr* group III. *Agr* group II has previously been associated with invasive disease and infective endocarditis [10], [28]. *Agr* groups are usually linked to CC affiliations; thus, all isolates within a given CC harbor the same *agr* alleles [14], [28]. An exception is CC45 that contains isolates belonging to *agr* groups I and IV with the latter being mainly observed in Asia and Australia [15]. Isolates that harbored *agr* group II alleles were found in CC5 and CC15, and the number of isolates in these CCs increased over time; thus, this might explain the increased prevalence of isolates that harbored *agr* group II alleles.

All isolates harbored alleles associated with either capsule type 5 or 8, but *cap*8 alleles were more prevalent. Since previous studies supported an association between serotype 5 and invasiveness [10], [29], we did not expect the *cap*5 positive isolates to decline over time. However, the reasoning used for explaining changes in the *agr* groups may apply here; i.e., the capsule serotypes were associated with CC affiliations.

Among the MSCRAMM genes, the prevalence of *sasG* increased over time. The gene prevalence mainly follows CC affiliation and the gene was previously shown to be associated with invasive disease [10]. Other MSCRAMM genes were harbored by a majority or all isolates, which is consistent with the high degree of conservation of these genes in the *S. aureus* genome [30]. As expected, the exfoliative toxin genes were rare, because staphylococcal exfoliative disease is not typically invasive. Only sporadic isolates harbored the PVL-toxin gene, which is not required in the pathogenesis of SAB disease; thus its detection was most likely accidental, reflecting the prevalence of PVL in the general population. PVL is more commonly associated with necrotizing pneumonia and severe primary skin infections [31], [32]. Overall, only occasional virulence genes displayed a decadal change in prevalence, and in those cases, the differences probably reflected their linkage to the CCs.

A slight increase over time was observed in blood cultures positive for *S. aureus* when comparing the first year of each decade. However, this study was not designed to investigate changes in SAB incidence. Moreover, the increased incidence may, in part, be explained by more frequent blood culture sampling.

A limitation of this study was the lack of clinical data. We could therefore not analyze molecular epidemiology in terms of the type of *S. aureus* infection; i.e., whether the bacteremia was community-onset, healthcare-associated, or nosocomial. Another limitation was that the isolates included originated from a limited geographical area.

Molecular epidemiological studies that focus on MSSA are needed. To our knowledge, this is the first study to examine changes in the MSSA population structure over a time period longer than 30 years. The MSSA population was genetically diverse and the prevalence of antibiotic resistance genes did not change during the study period. The results suggest minor molecular alterations in some predominating CCs, and consequently, in the prevalence of *cap* genes and *agr* alleles, which are associated with CC affiliations. It may be difficult to conclude whether these shifts occurred continuously over time, or represent temporary variations, because the various CCs comprised a limited number of isolates in each decade.

However, our findings of predominant CCs were similar to those of previous studies, and we did not find a single clone that was entirely predominant. Thus, it is reasonable to assume that host factors may be at least as important as molecular epidemiology for explaining the increasing incidence of SAB.
